# Indications and Limitations of Sirolimus in the Treatment of Vascular Anomalies—Insights From a Retrospective Case Series

**DOI:** 10.3389/fped.2022.857436

**Published:** 2022-05-23

**Authors:** Anna Karastaneva, Paolo Gasparella, Sebastian Tschauner, Roman Crazzolara, Gabriele Kropshofer, Manfred Modl, Andreas Pfleger, Ante Burmas, Mirjam Pocivalnik, Raphael Ulreich, Werner Zenz, Wolfgang Schwinger, Besiana P. Beqo, Christian Urban, Emir Q. Haxhija, Herwig Lackner, Martin Benesch

**Affiliations:** ^1^Division of Pediatric Hematology and Oncology, Department of Pediatric and Adolescent Medicine, Medical University of Graz, Graz, Austria; ^2^Department of Pediatric and Adolescent Surgery, Medical University of Graz, Graz, Austria; ^3^Department of Radiology, Medical University of Graz, Graz, Austria; ^4^Department of Pediatrics, Medical University of Innsbruck, Innsbruck, Austria; ^5^Division of Pediatric Pulmonology and Allergology, Department of Pediatric and Adolescent Medicine, Medical University of Graz, Graz, Austria; ^6^Division of Pediatric Cardiology, Department of Pediatric and Adolescent Medicine, Medical University of Graz, Graz, Austria; ^7^Pediatric Intensive Care Unit, Department of Pediatric and Adolescent Medicine, Medical University of Graz, Graz, Austria; ^8^Division of General Pediatrics, Department of Pediatric and Adolescent Medicine, Medical University of Graz, Graz, Austria; ^9^Global Clinical Scholars Research Training, Department of Postgraduate Medical Education, Harvard Medical School, Boston, MA, United States

**Keywords:** sirolimus, vascular anomalies, vincristine, Kasabach–Merritt phenomenon, central conducting lymphatic anomaly (CCLA), epithelioid hemangioendothelioma (EHE), COVID-19

## Abstract

**Background:**

Despite recent developments, the role of sirolimus in the heterogeneous spectrum of vascular anomalies is yet to be defined, in terms of indication, dosage, and therapy duration, recognizing both its potential and limitations.

**Methods:**

We retrospectively analyzed 16 children with vascular anomalies treated with sirolimus in two pediatric centers between 2014 and 2020 [male: *n* = 7, the median age at diagnosis: 4.6 months (range, 0–281.4)]. In addition, repetitive volumetric analyses of the vascular anomalies were performed when possible (11 cases).

**Results:**

Ten patients were diagnosed with vascular malformations and 6 with vascular tumors. The mean therapy duration was 27.2 months (range, 3.5–65). The mean sirolimus level was 8.52 ng/ml (range, 5.38–12.88). All patients except one with central conducting lymphatic anomaly responded to sirolimus, with the most noticeable volume reduction in the first 4–6 months. Additional administration of vincristine was needed in five patients with kaposiform hemangioendothelioma and yielded a response, even in cases, refractory to sirolimus monotherapy. As a single agent, sirolimus led to impressive improvement in a patient with another vascular tumor—advanced epithelioid hemangioendothelioma. Complicated vascular malformations required long-term sirolimus therapy. Side effects of sirolimus included mucositis and laboratory abnormalities. No major infectious episodes were recorded. An infant with COVID-19, diagnosed while on sirolimus therapy, presented with a mild course.

**Conclusion:**

In the current series, we reported limitations of sirolimus as monotherapy, addressing the need to redefine its indications, and explore combination regimens and multimodal treatment strategies. Tools for objective evaluation of response trends over time could serve as a basis for the establishment of future therapeutic algorithms.

## Introduction

The rarity and highly variable clinical features of vascular anomalies (VAs) pose a great challenge regarding diagnostic and therapeutic management. The individualized multidisciplinary approach required for optimal management of VA complicates the systematic data collection and the development of standardized treatment recommendations. Since the first reports on the successful use of mammalian target of rapamycin (mTOR) inhibition for the treatment of kaposiform hemangioendothelioma (KHE), sirolimus rapidly evolved from a compassionate use agent to an established therapeutic option for this entity (1, 2). Rapid resolution of Kasabach–Merritt phenomenon (KMP), a cause of significant morbidity and mortality in KHE, along with reduction of the mass effect and complete remissions even in cases refractory to other therapies was observed (1, 3–6). Furthermore, sirolimus seems to have a stabilizing effect on malignant vascular tumors such as advanced epithelioid hemangioendothelioma (EHE), but consistent data are still lacking (7, 8). In addition, mTOR inhibition was introduced in the treatment algorithm for vascular malformations with response rates ranging between 80 and 100% in patients with simple lymphatic malformations (LMs) (9, 10). Impressive clinical and radiological improvements were also observed in patients with generalized lymphatic anomaly (GLA), kaposiform lymphangiomatosis (KLA), and Gorham–Stout disease (GSD) (10–13). Treatment responses were also described in complex low flow malformations such as capillary-lymphatic- and capillary-lymphatic-venous malformations (CLM and CLVM) (2, 11, 14–16). In these entities, sirolimus is often applied as a tool to further facilitate sclerotherapy or resection (10, 11, 14). Data on the use of sirolimus in high flow vascular malformations are still scarce and not convincing (17). Recent reports suggest partial responses and potential benefits from mTOR inhibition as an adjuvant therapy before and following endovascular embolization for arteriovenous malformations (AVMs) (11, 18). It is hypothesized that heterogeneous responses of high flow malformations to sirolimus might be related to their molecular background (19). The scarcity of molecular substrate in AVM as a target for mTOR inhibitors would result in poor response and vice versa (20). The efficacy of sirolimus in AVM is being investigated in a prospective phase II study (21).

Despite these recent encouraging developments, the role of sirolimus in the heterogeneous spectrum of VA is yet to be defined, recognizing both its potential and limitations, and place as a part of a combination and/or multimodal therapy. In this retrospective series, we reviewed pediatric patients with various VA, treated with the mTOR inhibitor sirolimus.

## Patients and Methods

The subject of this retrospective analysis is patients diagnosed with VA from November 2014 to December 2020 who received systemic therapy with sirolimus in two pediatric centers. The VAs in our case series were categorized according to the classification of the International Society for the Study of Vascular Anomalies (ISSVA, 2018) (22).

**Figure 1:**
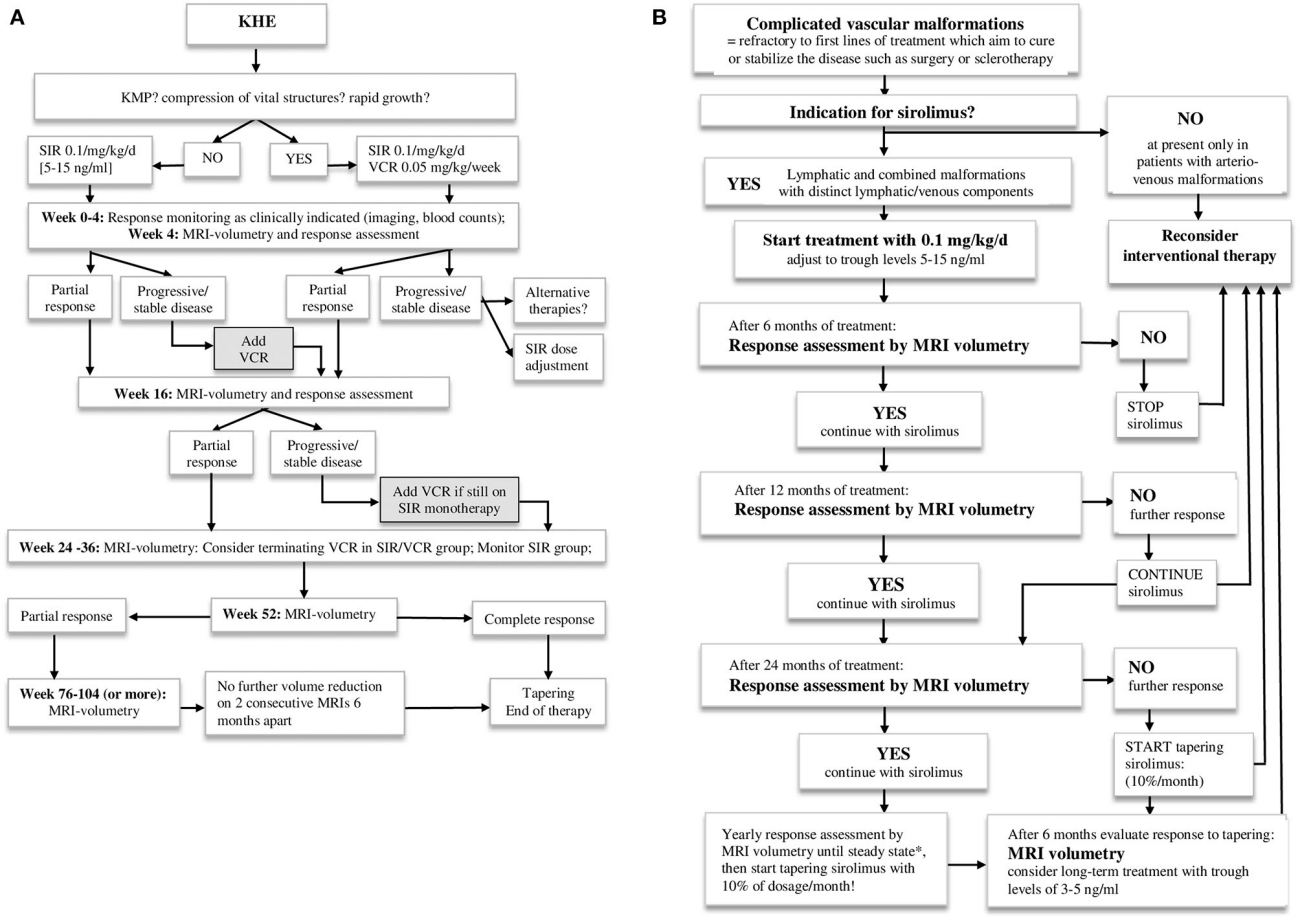
**(A)** Treatment approach for patients with kaposiform hemangioendothelioma. KHE, kaposiform hemangioendothelioma; KMP, Kasabach-Merritt phenomenon; SIR, sirolimus; VCR, vincristine. **(B)** Treatment approach for patients with complicated vascular malformations. *Steady state is defined as a stable disease in two consecutive MRT volumetries at least 6 months apart.

The indications for treatment with sirolimus, applied in these case series, included: KHE with or without KMP; EHE; LM and combined vascular malformations with a distinct lymphatic and/or venous component and overt or imminent organ dysfunction/disfigurement unlikely to be sufficiently reduced by upfront local/surgical treatment. Initially, all children received sirolimus at a dose of 0.1 mg/kg/d orally, divided into two daily doses, which was subsequently adjusted to target trough levels within the range of 5–15 ng/ml. Treatment intensification with vincristine was considered in patients with KHE and KMP and/or other aggravating symptoms or those refractory to sirolimus monotherapy. Interventional therapeutic options were considered in patients with vascular malformations at any point upon re-evaluation of response to sirolimus. The treatment schedules, we applied in our institutions, including targeted response-evaluation intervals, are described in Figures 1A,B.

Relevant previous or concomitant therapies were documented. In addition, all patients received oral *Pneumocystis jirovecii* prophylaxis with trimethoprim/sulfamethoxazole during treatment with sirolimus.

The response evaluation was performed by clinical examination, volumetric comparison of the VA, monitoring of laboratory parameters (blood counts and coagulation status), and reflecting the dynamics of pre-existing KMP or coagulopathy. The volumetric comparison was assessed by imaging studies [magnetic resonance tomography (MRT) and CT]. The Digital Imaging and Communications in Medicine (DICOM) images of the CT and MRT studies were loaded to a workstation running the 3D slicer open-source software (https://www.slicer.org/), version 4.7.0-2016-11-04r25501 (23). The tumor lesions were manually segmented by a pediatric radiologist in all relevant slices. After the procedure, volumes could be read out automatically with the “label statistics” module. Then, the volume of the VA was set in relation to the current body surface area (BSA) of the patient at each time point (VA volume/BSA, normalized VA volume). This parameter was used to assess the dynamics of VA over time in each case. For VA of the head and neck, the respective circumference was used instead of BSA due to the limited contribution of these regions to the BSA.

The time to response was defined clinically by the first documented reduction of symptoms or volume reduction on imaging. The outcome was classified into 4 categories—complete response (CR): the disappearance of the lesion and the symptoms; partial response (PR): improvement of the symptoms and volume reduction of the VA; stable disease (SD): no further progression; and progressive disease (PD): progression of the size of the VA or related symptoms while on sirolimus.

This retrospective analysis was approved by the institutional review boards of the two participating centers.

## Results

**Figure 2:**
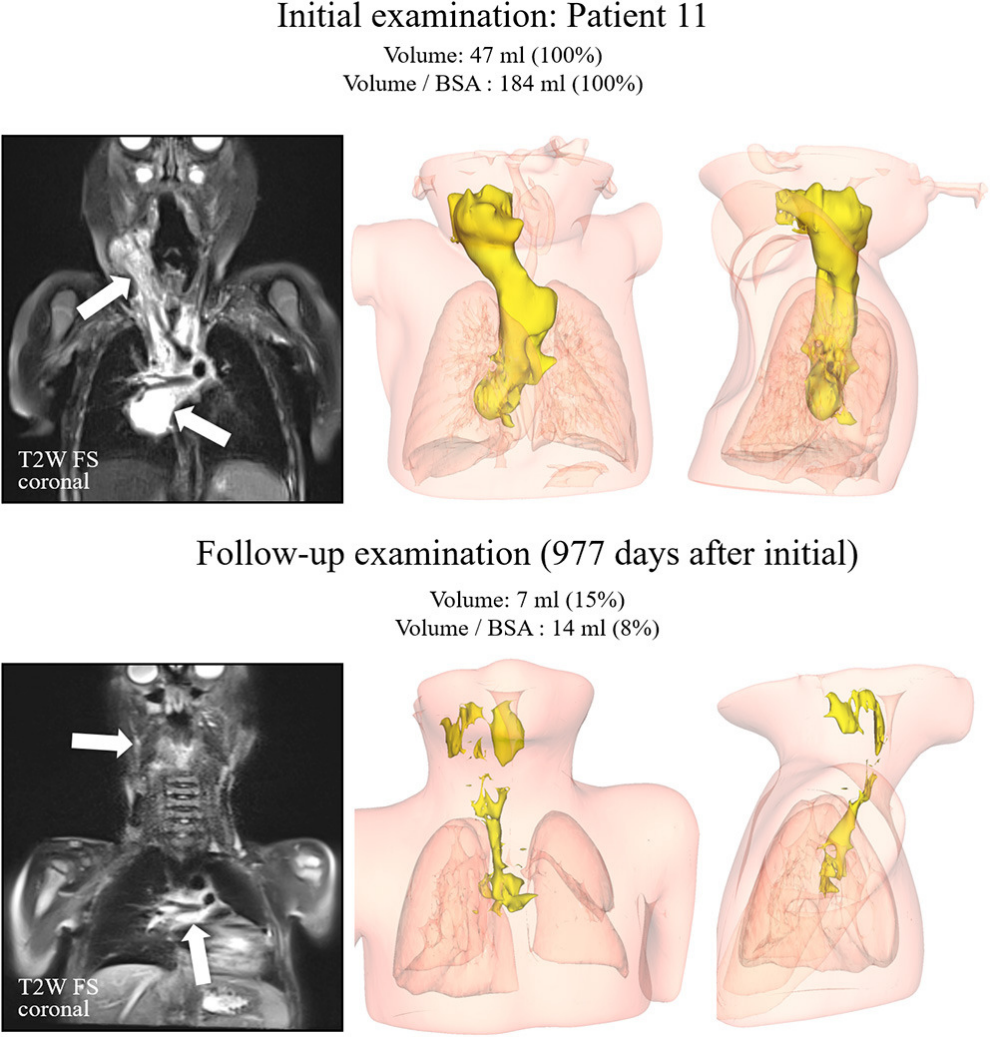
Patient #11. T2-weighted images with fat saturation in the coronal plane on the left side for both examinations. The kaposiform hemangioendothelioma demonstrated a high signal intensity (arrows) on both examinations, with substantial volume reduction over time. Corresponding 3D reconstructions in anteroposterior and mediolateral views are displayed in the middle and right columns.

A total of 16 pediatric patients (9 girls and 7 boys) were available for evaluation. Baseline characteristics are summarized in Table 1. The spectrum of VA consisted of 10 patients with vascular malformations [simple LM, *n* = 6, combined lymphatic-venous malformation (LVM), *n* = 1 and arteriolymphatic-venous malformation (ALVM), *n* = 1, CLAPO-syndrome (lower lip capillary malformation/face and neck LM asymmetry and partial overgrowth), *n* = 1 and CLOVE-syndrome (congenital lipomatous overgrowth, vascular malformations, epidermal nevi, skeletal, and spinal abnormalities), *n* = 1] and six patients with vascular tumors (KHE, *n* = 5; advanced EHE, *n* = 1). Three out of 5 cases with KHE had KMP at diagnosis (Table 1). Biopsies were performed at diagnosis in 8 of 16 children (50%); in the remaining 8, the diagnosis was based on characteristic clinical and imaging features of VA. In patients #1–#5, #9, and #14 the diagnosis was based on clinical features, a biopsy was performed in patients #6–#8, #10, #12, #13, #15, and #16. One patient (#11) presented with a non-chylous pericardial effusion and an extensive mediastinal mass of vascular origin on imaging, exhibiting features of KHE with a mild transient coagulopathy and no signs of KMP. Unfortunately, the biopsy had to be interrupted before a tissue sample could be obtained because of life-threatening bleeding (Figure 2).

**Table 1:** Patient characteristics and clinical course.

**No**.	**Age (months)**	**♀♂**	**Diagnosis**	**Site**	**Biopsy**	**SIR duration (months)**	**SIR mean trough level (ng/ml)**	**VCR duration (months)**	**Time to response (months)**	Other interventions^*^	**Outcome**	**Follow-up (months)**
1	0	**♀**	LM	Cervicofacial	–	33	5.78		0.75	–	PR	Ongoing
2	2.5	**♂**	LM	Cervicofacial	–	11	5.53		0.5	–	PR	Ongoing
3	0.75	**♀**	CCLA	Lung	–	3.5	12.01		n/a	Multiple	PD	n/a
4	0	**♀**	CLAPO	Cervicofacial, skull base	–	14	7.53		1	OK432, tracheostomy	PR	18
5	0	**♀**	CLOVES	Thorax	–	9	12.88		0.25	–	PR	Ongoing
6	4.2	**♂**	GSD	Skull base	+	58	8.60		0.5	Pamidronate	PR	Ongoing
7	127.2	**♂**	GLA	Lung, pericardium	+	50	9.63		2	Steroids	PR	Ongoing
8	179	**♀**	GLA	Lung, spleen, retroperit.	+	9	5.38		2	–	PR	Ongoing
9	37.2	**♂**	LVM	Orbita	–	36	8.00		1	–	PR	10
10	218.4	**♀**	ALVM	Cervicofacial, skull base	+	30	9.44		1.5	2 × Surgery	PR	Ongoing
11	5	**♂**	KHE	Neck, mediastinum	–^***^	27	9.03	9	4.5	Pericardial fenestration	PR	5
12	14	**♂**	KHE+KMP	Thigh	+	16	9.81	3	0.75	Embolization	CR	64
13	5	**♀**	KHE	Liver	+	24	9.82	3	0.5	Multiple	PR	51
14	3	**♀**	KHE+KMP	Cervical	–	16	6.42	6	1	–	CR	8
15	2	**♀**	KHE +KMP	Thigh	+	33	9.05	6	1.5	–	CR	15
16	134.4	**♂**	EHE	Liver, lung	+	65	7.48		3	–	PR	Ongoing
Median	4.6							6	1			
Mean^**^						27.2	8.52					
*N (%)*					8/16 (50%)					8/16 (50%)		

*Age, age at the beginning of sirolimus therapy; SIR, sirolimus therapy; VCR, vincristine intensification; LM, lymphatic malformation; CCLA, central conducting lymphatic anomaly; CLAPO, lower lip capillary malformation/face and neck LM asymmetry and partial overgrowth-syndrome; CLOVES, congenital lipomatous overgrowth, vascular malformations, epidermal nevi, skeletal and spinal abnormalities-syndrome; GSD, Gorham–Stout disease; GLA, generalized lymphatic anomaly; LVM, lymphatic-venous malformation; ALVM, arterio-lymphatic-venous malformation; KHE, kaposiform hemangioendothelioma; KMP, Kasabach–Merritt phenomenon; EHE, epithelioid hemangioendothelioma*.

* *Interventions performed prior to or during the course of sirolimus therapy*.

** *Mean was calculated for the parameters with normal distribution*.

*** *Biopsy attempted but failed to deliver a tissue sample due to a severe life-threatening intraoperative bleeding*.

**Figure 3:**
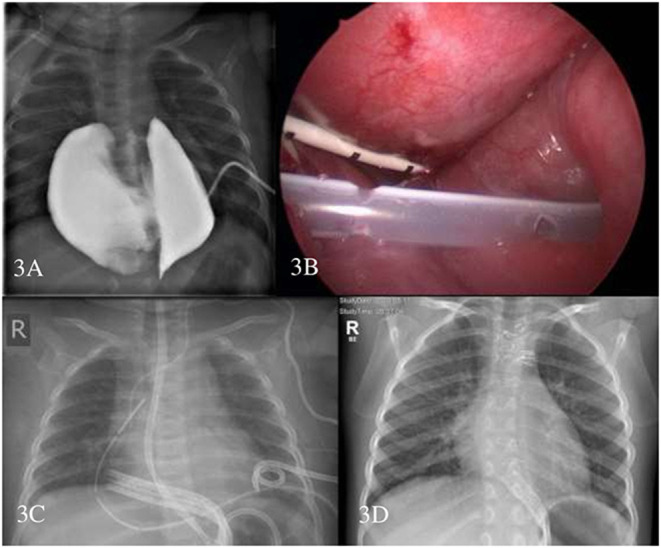
Patient #3. Contrast filling of the intrathoracic central conducting lymphatic anomaly (CCLA) through the pigtail catheter to depict the anatomic relationship **(A)** thoracoscopic placement of the Denver drain—pigtail drain is seen **(B)** chest X-ray with multiple drains **(C)** and chest X-ray 1-year after sternotomy and surgical resection of CCLA **(D)**.

At the start of sirolimus therapy, the median age was 4.6 months (range, 0–218.4 months). Ten patients (62.5%) received upfront sirolimus therapy, and 6 patients (37.5%) had already undergone previous treatments. The mean trough level of sirolimus was 8.52 ng/ml (5.38–12.88) (Table 1). All patients except one responded to sirolimus; three (18.75%) achieved CR and 12 (75%) PR. One patient (#3) developed PD of a giant bilateral intrathoracic cystic lesion/chylous effusion in the context of a central conducting lymphatic anomaly (CCLA) during treatment with sirolimus. Following the failure of multiple interventions, namely, cyst drainage, sclerotherapy, and an attempt to shunting into the abdominal cavity, a thoracotomy with surgical resection and tapering of the bilateral cysts led to the resolution of the disease, and no further therapy was required (Figure 3).

**Figure 4:**
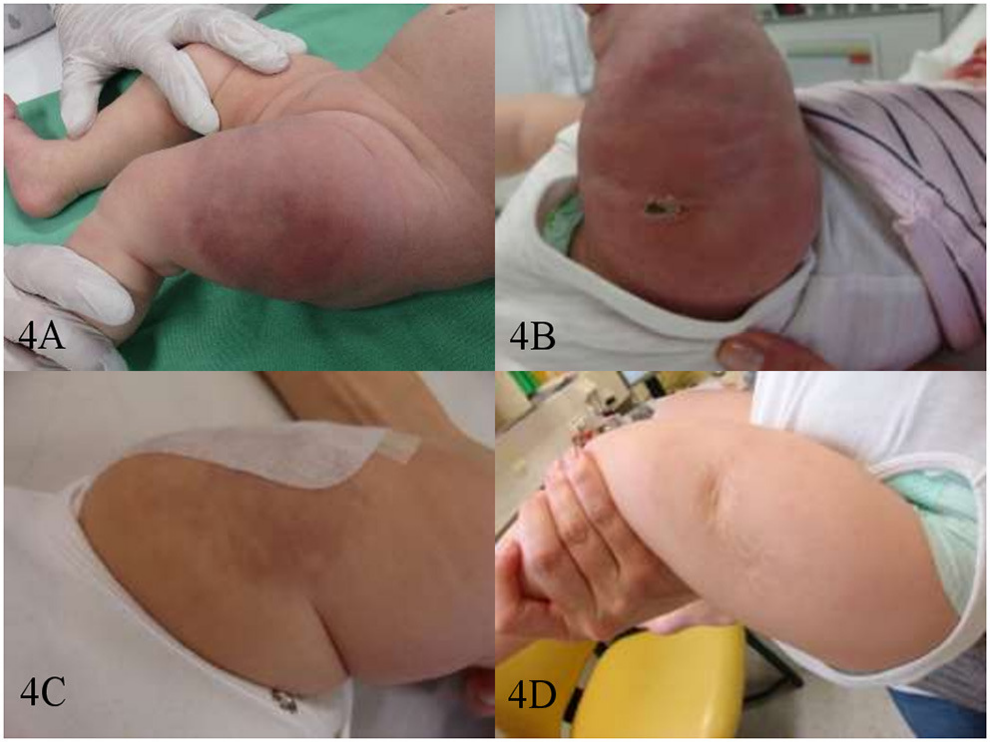
Patient #15. Kaposiform hemangioendothelioma of the left thigh before therapy **(A)** on day 20 of sirolimus monotherapy—after 1st biopsy **(B)** on day 14 of sirolimus/vincristine combination therapy—after the 2nd biopsy **(C)** and after 1 year of therapy-−6 months of sirolimus/vincristine, and 6 months of consecutive sirolimus monotherapy **(D)**.

The median time of response in patients with PR and CR was 1 month (0.5–4.5). Patients with KHE (*n*=5) received treatment intensification with weekly vincristine (0.05 mg/kg/week) concurrently to sirolimus. Due to the bulky disease, the severe KMP, and the critical location in 3 patients (#12, #13, and #14), the intensification therapy was started at diagnosis. Whereas, in patients #11 and #15 the intensification therapy was started after initial refractoriness to sirolimus and PD (Figures 2, 4). The latter two children had monotherapy with sirolimus for 3.8 and 1 month, respectively, before vincristine was added. The response was observed <1 month later. KMP in patients with KHE resolved in all cases within the first few days after initiation of therapy (sirolimus or combination). Once KMP disappeared, and the tumor mass was observed to reduce in a steady-state mode, vincristine could be tapered and discontinued. We did not observe a rebound phenomenon following the usage of this protocol. Apart from the requirement for a central venous line, vincristine at this dosage level was tolerated without any side effects.

The mean duration of sirolimus therapy was 27.2 months (3.5–65). Eight patients are still under care, and 8 others are already off treatment (follow-up periods are presented in Table 1). Tapering was started after the best response was achieved and confirmed in 2 consecutive imaging studies. In one case (#15), sirolimus was discontinued and had to be restarted after 12 months because of a relapse, resulting in a second CR. The patient with CLAPO-syndrome, who initially responded well to sirolimus, experienced PD after discontinuation of sirolimus upon parents' wish. She is still on observation due to her parents' concerns regarding potential infectious complications in the setting of her tracheostomy.

**Figure 5:**
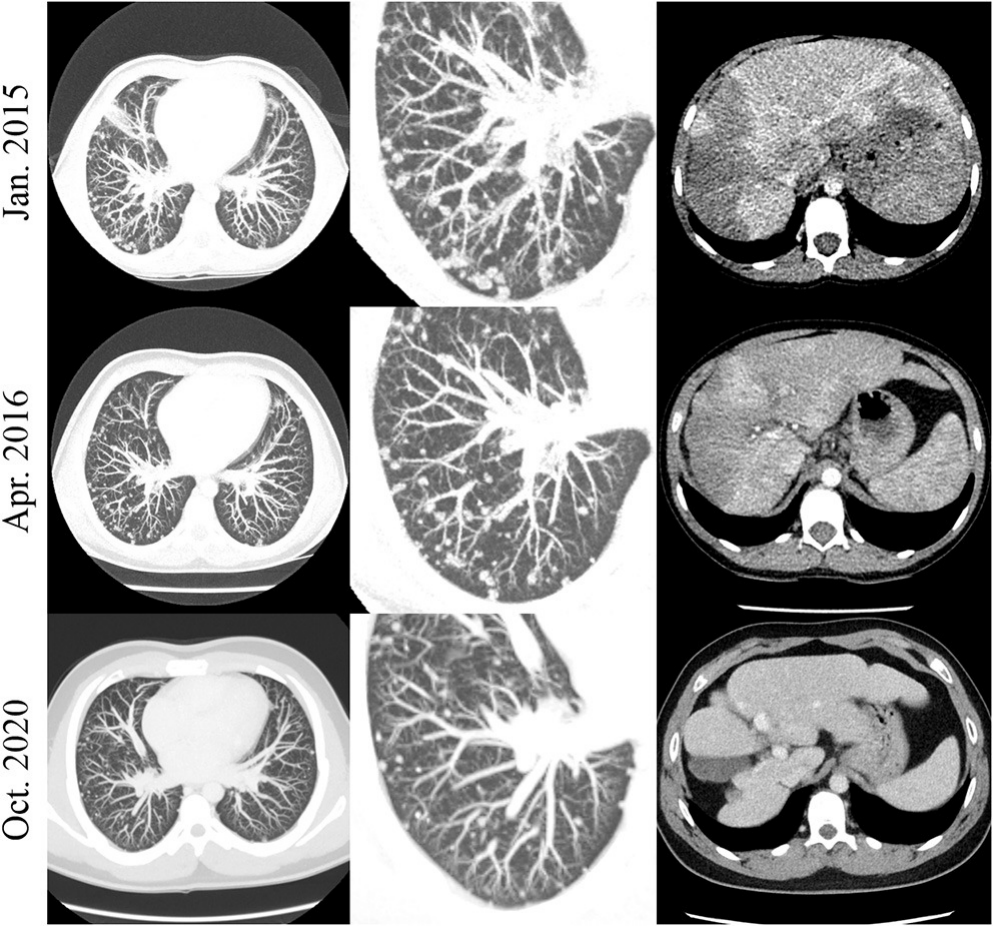
Patient #16. Exemplary chest (left and middle row) and abdomen (right row) CT slices in patient #16. A decrease in the amount and size of the numerous nodular lung lesions is noted over time. The initially hypodense liver lesions were involuted over the course of the examinations.

The patient with EHE (#16) has the most prolonged therapy duration after an excellent initial response and continues to receive sirolimus to maintain PR without any treatment-related complications (Figure 5).

**Figure 6:**
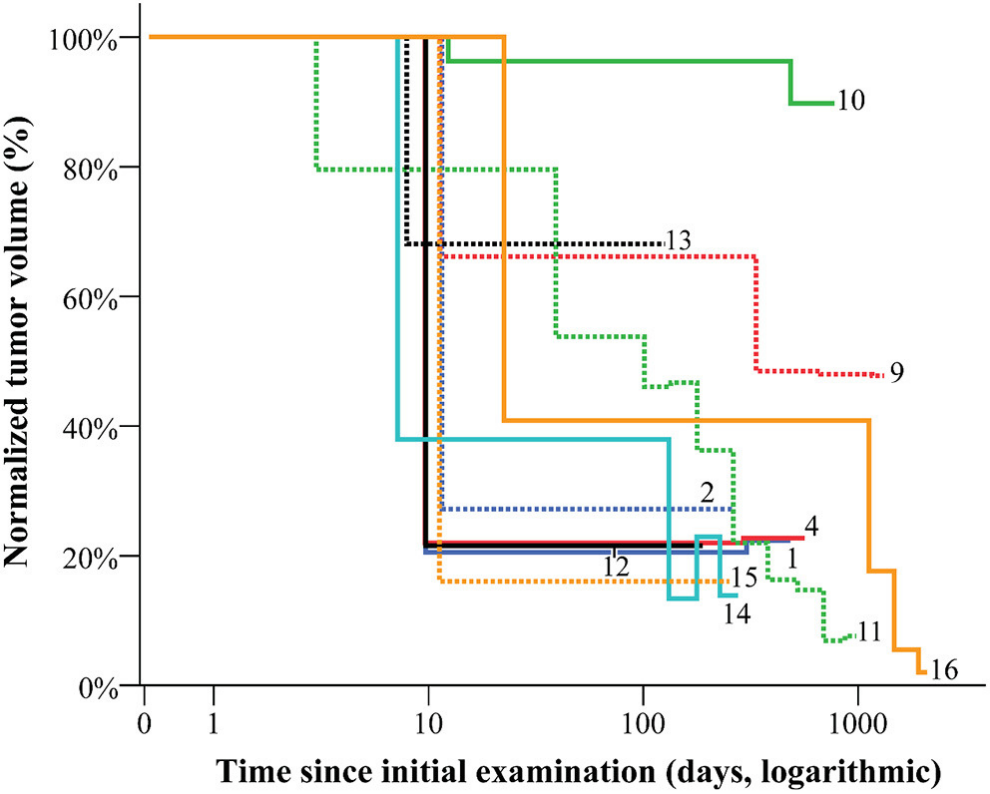
The volume of vascular anomalies during therapy and early follow-up. The normalized volume of vascular anomalies was calculated considering the factor of patients' growth when assessing the response to therapy. The most remarkable volume reductions are to be detected within the first 4–6 months of therapy.

In 11 of 16 cases, the response to therapy could be demonstrated through serial imaging-based volumetric studies of the VA, considering the change of the BSA of the patient (or circumference of the affected region for VA of the head and neck) between the examinations (normalized VA volume, Figure 6). The most remarkable volume reductions were observed within the first 4–6 months of therapy in all entities. Yet, primarily non-resectable vascular malformations prove to require long-term therapy to sustain PR (Table 1 and Figure 6). In diffuse or destructive lesions (GSD, GLA, and CLOVES-syndrome), exact volumetry of the VA was not possible. The patient with CCLA was not included as the volume of the VA did not change. The EHE patient from our series showed a response trend similar to vascular malformations.

Both sirolimus monotherapy and the combination with vincristine were generally well-tolerated. Recurrent mucositis in 2 cases was resolved with improved oral hygiene and dose adjustment. In 4 children, mild infections affecting the airways and gastrointestinal tract were observed. However, no tendency was noticed toward an increased susceptibility to infections or major infectious complications. One patient (#2) was diagnosed with COVID-19, but as the clinical course was uncomplicated, sirolimus could be restarted within 2 weeks. Generally, sirolimus was temporarily discontinued or continued in reduced dosage during infectious episodes.

In 4 cases, transient hypertriglyceridemia was detected at the beginning of the treatment. One patient manifested an unexplained asymptomatic creatine kinase-myocardial band elevation, which normalized spontaneously within 2 months. Interestingly, 8 of our 16 patients developed striking microcytosis without iron deficiency, mostly with normal hemoglobin levels during sirolimus therapy (similar to that observed in the ß-thalassemia trait). To this end, some of the young children who had not had blood counts analyzed before starting sirolimus therapy underwent hemoglobin electrophoresis and genetic testing to rule out the ß-thalassemia trait.

## Discussion

Sirolimus proved to be an effective therapeutic option, especially in life-threatening vascular tumors, and it alleviates the disease burden in patients with vascular malformations, corroborating previous results reported by our group (3, 15, 16). However, in the current case series, treatment intensification with vincristine was required in 5 patients, indicating limitations of sirolimus as a single therapeutic agent.

Vincristine, in combination with steroids, has historically been a first-line pharmacologic therapy for KHE with KMP. Steroid alone was indicated for KHE without KMP but requiring intervention (24). The first reports on sirolimus in VA are derived from experience in critical clinical situations of vincristine-refractory VA with KMP, where the mTOR inhibitor seemed to yield a rapid response within the first week of treatment (1–3, 25). Similarly, Tribolet et al. added sirolimus to the treatment regimen of a patient with KHE and severe KMP not responding to an 8-day course of propranolol, steroid, and vincristine and observed rapid resolution of the lesion (26). A similar experience was reported by Cashell et al., where the initial regimen did not include propranolol (5). A recent comprehensive literature review compared vincristine to sirolimus in KHE management with or without KMP, proving sirolimus superiority over vincristine (27). In another retrospective analysis of 30 patients with KHE, ~50% presenting with KMP, sirolimus yielded better results than monotherapy with steroid, vincristine, or propranolol (28). Due to the favorable response rates obtained by sirolimus as a single agent, its safety profile, and the oral formulation, we applied sirolimus upfront in 2 cases. We added vincristine after monotherapy failed to induce a proper response in KHE. The indication for intensification for patient #11 was a pericardial effusion that repeatedly evolved into a clinical emergency. Following the biopsy, patient #15 also experienced an increase in tumor volume while on sirolimus monotherapy. In all cases, a response was obtained by adding vincristine, which could be withdrawn as soon as a stable decline in tumor/effusion volume was notable. We used vincristine and sirolimus concurrently upfront in another KHE patient with an imminent airway compression and KMP (#14) and another 2 children with multiple previous treatments (#12 and #13). Treatment intensification—in our series—with vincristine, might be indicated in cases refractory to single-agent sirolimus. In our patients, a beneficial effect on the tumor mass was observed. As we included only 3 cases with KMP and in all of them the thrombocytopenia resolved rapidly under or before intensification, it is difficult to hypothesize any additional impact of vincristine, regarding KMP. Brill et al. suggest embolization of KHE before sirolimus might induce a more rapid resolution of KMP—within a median of 7 days, compared to 3 months for sirolimus alone (29). We observed a similar course (Table 1, Patient #12) after 2 embolizations, however, followed by sirolimus and concomitant vincristine. Another combination regimen—sirolimus and prednisolone, demonstrated benefits for patients with KHE and KMP compared to sirolimus alone in a recent randomized clinical trial, confirming data from a previous retrospective study (30, 31). We applied prednisolone successfully in addition to sirolimus just in one case with GLA (#7), already in PR, to accelerate the resolution of the pericardial effusion.

Our observations of initial PD under sirolimus monotherapy in 3 cases (#3, #11, and #15) might be attributed to disease-related factors (effusions, abnormal wound healing due to abnormal vascularization), overlapping with those of sirolimus' well-known side effects—susceptibility to effusions/peripheral edema and compromised wound healing. Patient #15 had initial progression as mTOR inhibition was introduced soon after biopsy, probably compromising the wound healing process (Figure 4). Data regarding the development of effusions during sirolimus therapy are controversial. Ricci et al. report the favorable response of effusions to sirolimus monotherapy in patients with GLA (32). In contrast, all patients in our series, presenting with effusions (*n* = 3), required additional intervention—vincristine (#11 for PD), surgery (#3 for PD), or a short course of steroid (#7 already in PR with sirolimus). Subsequently, 2 of them could be successfully managed with sirolimus alone; the third one, diagnosed with CCLA (#3), required no further therapy after surgery. The treatment of choice for this entity is still surgical, but partial responses to sirolimus are reported in very few cases (32). Stacchiotti et al. also observed a more unsatisfactory outcome in a subset of patients with advanced EHE and effusions on sirolimus monotherapy. However, sirolimus appeared to generally stabilize the advanced/progressive disease within this cohort (7). Even so, our patient with advanced EHE (#16) achieved a sustained PR. Still receiving sirolimus, his quality of life is excellent, including intensive sports activities in everyday life. Despite therapy duration of more than 5 years (patient #16), no relevant side effects have occurred so far. Yet, the mean trough level, in this case, is lower (7.48 ng/ml) than the target levels suggested by Stacchiotti et al. (15–20 ng/ml), perhaps explaining the better tolerability without therapy interruptions (7).

Similarly, all our patients with complicated vascular malformations, who responded well to sirolimus monotherapy, need timely undefined, but presumably much longer treatment with sirolimus than those with KHE. It is still unclear if targeting lower trough levels of sirolimus to reduce possible long-term side effects would suffice to sustain the achieved PR. We reduced the dose when no further change in the volume of the lesion occurred within 1 year under steady therapeutic trough levels. The same was applied to patients with complete resolution of the lesions. However, sufficient evidence for the safety of this approach cannot be derived from our data due to the limited number of cases in our series.

The tolerability of sirolimus compared to other treatment modalities for VA seems to be excellent. However, immunosuppression and possible long-term consequences, especially in young children, where fatalities due to infections have already been reported, require considerations about the appropriate duration of therapy and the need for dose adjustments when continuous treatment is indicated (33). Regarding infectious complications, our series shows contradictory yet allegeable observations. Four patients in our cohort (#4, #9, #11, and #15) had frequent mild infections, presumably due to the immunosuppressive effect of mTOR inhibition. In another 2 cases (#6 and #7), after therapy with sirolimus was initiated, mTOR inhibition resulted in complete resolution of chronic infections (*Streptococcus pneumoniae*—meningitis and Campylobacter—pericarditis). Ying et al. reported two cases of fatal respiratory infections in young infants under sirolimus for VA, pointing to this age as potentially more vulnerable to the immunosuppressive properties of this drug (33).

In the setting of the COVID-19 pandemic, additional attention toward chronically immunocompromised patients was raised. One of the patients from our series (#2) was diagnosed with COVID-19 at the age of 4 months. The clinical course, in this case, was mild compared to the infants mentioned with fatal respiratory infections under sirolimus therapy. A comparably benign course of COVID-19 was also reported in a 13-year-old patient receiving mycophenolate mofetil and sirolimus after kidney transplantation (34). Omarjee et al. recently discussed the immunomodulatory properties of sirolimus on T-cells, responsible for the hyperinflammatory state in patients with the severe clinical course of COVID-19, as a possible explanation (35). On the other hand, a report by Meziyerh et al. describes severe COVID-19 complications in an adult kidney transplant recipient, receiving everolimus, speculating on impaired mucosal function under mTOR inhibition and drug interactions as possible triggers (36).

The issues regarding possible therapy complications and effects of dose adjustments seem particularly relevant in situations with long-term therapy and SD where the further benefit of continuous sirolimus administration is not expected or difficult to assess (patients #6 and #16). Yet, a progression after discontinuation could have life-threatening consequences. Therefore, further studies on the objective dynamics of treatment response by repetitive volumetry of the VA could facilitate clinical decision-making. We utilized BSA-adapted volumetry, reflecting the patients' growth, which might be a more reliable method for volumetric assessment than the simple comparison of the absolute volumes of the VAs, especially in young children and long-term therapies. The potential of this method needs to be explored in larger patient collectives.

Encouraging data for a successful restart of sirolimus from Chelliah et al., Stacchiotti et al., and Adams et al. supports our observation of patient #15, who responded well to the second course of sirolimus for re-activation of her KHE (7, 11, 18). Failure to achieve response by re-challenge with sirolimus in relapsed VA after successful initial therapy has not been reported so far. Time to initial clinical and/or radiological response, effects of tapering, treatment intervals, and treatment restarting in the disease course are parameters that may be used to adapt the current treatment recommendations.

## Conclusion

After implementing sirolimus for the treatment of KHE in 2010, the dynamic development in this field resulted in an increasing number of indications for mTOR inhibition within the heterogeneous group of VA. Along with the indisputable benefits, the expanding experience raises awareness of possible risks and limitations as well. The analysis of treatment results in our case series, and, in particular—those of therapeutic failures to sirolimus monotherapy provided insights on a potentially effective combination regimen with vincristine. The increasing options for beneficial combinations with other second agents such as steroids or interventional modalities require further specifying the indications for these and the role of sirolimus in the multimodal therapeutic approach for VA. We also addressed questions concerning supportive care and management strategies, especially in cases requiring long-term therapy with sirolimus. The volumetric studies, reflecting response trends over time, might facilitate future recommendations for dosage and therapy duration. The implementation of such tools could serve as a basis for future treatment algorithms.

## Data Availability Statement

The raw data supporting the conclusions of this article will be made available by the authors, without undue reservation.

## Ethics Statement

The studies involving human participants were reviewed and approved by Ethics Committee Medical University Graz (EK 32-236 ex19/20). Written informed consent from the participants' legal guardian/next of kin was not required to participate in this study in accordance with the national legislation and the institutional requirements.

## Author Contributions

AK participated in the clinical care, coordination, and patient recruitment, conceptualized and drafted the manuscript, the table, and the algorithms. PG was involved in the surgical care and recruited patients, edited the references, and prepared the manuscript for submission. ST interpreted the imaging studies, performed the volumetric studies, created the graph, and selected and edited the images for the figures. EQH was a senior surgical advisor, conceptualized the figures, added insights regarding cases with vascular malformations, conceptualized the algorithm for patients with complicated vascular malformations, and reviewed and revised the manuscript. BPB collected data, reviewed, adapted, and formatted the figures, and revised the manuscript. RC and GK guided the clinical care for the patients in one of the centers, recruited patients, and collected data. WS, MM, AP, AB, MP, RU, and WZ were involved in establishing therapeutic concepts for challenging patients as a part of the multidisciplinary team, participated in recruitment, and data collection. HL was a senior medical advisor, conceptualized the study, summarized the major contributions, and reviewed the manuscript. CU and MB were senior clinical advisors and critically reviewed and revised the manuscript and the final version. All authors approved the final manuscript as submitted and agree to be accountable for all aspects of the work.

## Funding

This study was financially supported by Land Steiermark (Office of the Regional Government of Styria, Department of Health Care and Science, Unit Science and Research, Austria).

## Conflict of Interest

The authors declare that the research was conducted in the absence of any commercial or financial relationships that could be construed as a potential conflict of interest.

## Publisher's Note

All claims expressed in this article are solely those of the authors and do not necessarily represent those of their affiliated organizations, or those of the publisher, the editors and the reviewers. Any product that may be evaluated in this article, or claim that may be made by its manufacturer, is not guaranteed or endorsed by the publisher.

## References

[1] Blatt J Stavas J Moats-Staats B Woosley J Morrell DS. Treatment of childhood kaposiform hemangioendothelioma with sirolimus. Pediatr Blood Cancer. (2010) 55:1396–8. 10.1002/pbc.22766 20730884

[2] Hammill AM Wentzel M Gupta A Nelson S Lucky A Elluru R. Sirolimus for the treatment of complicated vascular anomalies in children. Pediatr Blood Cancer. (2011) 57:1018–24. 10.1002/pbc.23124 21445948

[3] Jahnel J Lackner H Reiterer F Urlesberger B Urban C. Kaposiform hemangioendothelioma with Kasabach-Merritt phenomenon: from vincristine to sirolimus. Klin Padiatr. (2012) 224:395–7. 10.1055/s-0032-1323823 23070861

[4] Chinello M Di Carlo D Olivieri F Balter R De Bortoli M Vitale V. Successful management of kaposiform hemangioendothelioma with long-term sirolimus treatment: a case report and review of the literature. Mediterr J Hematol Infect Dis. (2018) 10:e2018043. 10.4084/mjhid.2018.043 30002799 PMC6039087

[5] Cashell J Smink GM Helm K Xavier F. Kaposiform hemangioendothelioma with Kasabach-Merritt phenomenon in an infant: successful treatment with prednisolone, vincristine, and addition of sirolimus. Pediatr Blood Cancer. (2018) 65:e27305. 10.1002/pbc.27305 30070028

[6] Wang H Duan Y Gao Y Guo X. Sirolimus for vincristine-resistant kasabach-merritt phenomenon: report of eight patients. Pediatr Dermatol. (2017) 34:261–5. 10.1111/pde.13077 28198567

[7] Stacchiotti S Provenzano S Dagrada G Negri T Brich S Basso U. Sirolimus in advanced epithelioid hemangioendothelioma: a retrospective case-series analysis from the italian rare cancer network database. Ann Surg Oncol. (2016) 23:2735–44. 10.1245/s10434-016-5331-z 27334221

[8] Engel ER Cournoyer E Adams DM Stapleton S. A retrospective review of the use of sirolimus for pediatric patients with epithelioid hemangioendothelioma. J Pediatr Hematol Oncol. (2020) 42:e826–9. 10.1097/MPH.0000000000001643 31714437

[9] Ozeki M Asada R Saito AM Hashimoto H Fujimura T Kuroda T. Efficacy and safety of sirolimus treatment for intractable lymphatic anomalies: a study protocol for an open-label, single-arm, multicenter, prospective study (SILA). Regen Ther. (2019) 10:84–91. 10.1016/j.reth.2018.12.001 30705924 PMC6348766

[10] Wiegand S Wichmann G Dietz A. Treatment of lymphatic malformations with the mTOR inhibitor sirolimus: a systematic review. Lymphat Res Biol. (2018) 16:330–9. 10.1089/lrb.2017.0062 29924669

[11] Adams DM Trenor CC III Hammill AM Vinks AA Patel MN Chaudry G. Efficacy and safety of sirolimus in the treatment of complicated vascular anomalies. Pediatrics. (2016) 137:e20153257. 10.1542/peds.2015-3257 26783326 PMC4732362

[12] Cramer SL Wei S Merrow AC Pressey JG. Gorham-stout disease successfully treated with sirolimus and zoledronic acid therapy. J Pediatr Hematol Oncol. (2016) 38:e129–32. 10.1097/MPH.0000000000000514 26886375

[13] Nozawa A Ozeki M Kuze B Asano T Matsuoka K Fukao T. Gorham-stout disease of the skull base with hearing loss: dramatic recovery and antiangiogenic therapy. Pediatr Blood Cancer. (2016) 63:931–4. 10.1002/pbc.25886 26713883

[14] Hammer J Seront E Duez S Dupont S Van Damme A Schmitz S. Sirolimus is efficacious in treatment for extensive and/or complex slow-flow vascular malformations: a monocentric prospective phase II study. Orphanet J Rare Dis. (2018) 13:191. 10.1186/s13023-018-0934-z 30373605 PMC6206885

[15] Vlahovic AM Vlahovic NS Haxhija EQ. Sirolimus for the treatment of a massive capillary-lymphatico-venous malformation: a case report. Pediatrics. (2015) 136:e513–6. 10.1542/peds.2014-3469 26148957

[16] Lackner H Karastaneva A Schwinger W Benesch M Sovinz P Seidel M. Sirolimus for the treatment of children with various complicated vascular anomalies. Eur J Pediatr. (2015) 174:1579–84. 10.1007/s00431-015-2572-y 26040705

[17] Triana P Dore M Cerezo VN Cervantes M Sanchez AV Ferrero MM. Sirolimus in the treatment of vascular anomalies. Eur J Pediatr Surg. (2017) 27:86–90. 10.1055/s-0036-1593383 27723921

[18] Chelliah MP Do HM Zinn Z Patel V Jeng M Khosla RK. Management of complex arteriovenous malformations using a novel combination therapeutic algorithm. JAMA Dermatol. (2018) 154:1316–9. 10.1001/jamadermatol.2018.3039 30326494 PMC6248124

[19] Gabeff R Boccara O Soupre V Lorette G Bodemer C Herbreteau D. Efficacy and tolerance of sirolimus (rapamycin) for extracranial arteriovenous malformations in children and adults. Acta Derm Venereol. (2019) 99:1105–9. 10.2340/00015555-3273 31386166

[20] Duran-Romero AJ Hernandez-Rodriguez JC Ortiz-Alvarez J Dominguez-Cruz JJ Monserrat-Garcia MT Conejo-Mir Sanchez J. Efficacy and safety of oral sirolimus for high-flow vascular malformations in real clinical practice. Clin Exp Dermatol. (2022) 47:57–62. 10.1111/ced.14841 34240451

[21] Prospective Evaluation of the Efficacy of Sirolimus (Rapamune®) in the Treatment of Severe Arteriovenous Malformations (MAV-RAPA). Available online at: ClinicalTrials.gov Identifier: NCT02042326 (accessed February 1, 2921).

[22] Wassef M Borsik M Cerceau P Faucon B Laurian C Le Clerc N. [Classification of vascular tumours and vascular malformations. Contribution of the ISSVA 2014/2018 classification]. Ann Pathol. (2021) 41:58–70. 10.1016/j.annpat.2020.11.004 33309330

[23] Fedorov A Beichel R Kalpathy-Cramer J Finet J Fillion-Robin JC Pujol S. 3D Slicer as an image computing platform for the Quantitative Imaging Network. Magn Reson Imaging. (2012) 30:1323–41. 10.1016/j.mri.2012.05.001 22770690 PMC3466397

[24] Drolet BA Trenor CC 3rd Brandao LR Chiu YE Chun RH Dasgupta R et al. Consensus-derived practice standards plan for complicated Kaposiform hemangioendothelioma. J Pediatr. (2013) 163:285–91. 10.1016/j.jpeds.2013.03.080 23796341

[25] MacFarland SP Sullivan LM States LJ Bailey LC Balamuth NJ Womer RB. Management of refractory pediatric kaposiform hemangioendothelioma with sirolimus and aspirin. J Pediatr Hematol Oncol. (2018) 40:e239–42. 10.1097/MPH.0000000000001046 29240034

[26] Tribolet S Hoyoux C Boon LM Cheruy C Demarche M Jamblin P. A not so harmless mass: kaposiform hemangioendothelioma complicated by a Kasabach-Merritt phenomenon. Arch Pediatr. (2019) 26:365–9. 10.1016/j.arcped.2019.06.003 31353149

[27] Peng S Yang K Xu Z Chen S Ji Y. Vincristine and sirolimus in the treatment of kaposiform haemangioendothelioma. J Paediatr Child Health. (2019) 55:1119–24. 10.1111/jpc.14370 30604513

[28] Ji Y Chen S Li L Yang K Xia C Li L. Kaposiform hemangioendothelioma without cutaneous involvement. J Cancer Res Clin Oncol. (2018) 144:2475–84. 10.1007/s00432-018-2759-5 30293120 PMC11813282

[29] Brill R Uller W Huf V Muller-Wille R Schmid I Pohl A. Additive value of transarterial embolization to systemic sirolimus treatment in kaposiform hemangioendothelioma. Int J Cancer. (2021) 148:2345–51. 10.1002/ijc.33406 33231291

[30] Ji Y Chen S Xiang B Li K Xu Z Yao W. Sirolimus for the treatment of progressive kaposiform hemangioendothelioma: a multicenter retrospective study. Int J Cancer. (2017) 141:848–55. 10.1002/ijc.30775 28486787

[31] Ji Y Chen S Zhou J Yang K Zhang X Xiang B. Sirolimus plus prednisolone vs sirolimus monotherapy for kaposiform hemangioendothelioma: a randomized clinical trial. Blood. (2022) 139:1619–30. 10.1182/blood.2021014027 35030255

[32] Ricci KW Hammill AM Mobberley-Schuman P Nelson SC Blatt J Bender JLG. Efficacy of systemic sirolimus in the treatment of generalized lymphatic anomaly and Gorham-Stout disease. Pediatr Blood Cancer. (2019) 66:e27614. 10.1002/pbc.27614 30672136 PMC6428616

[33] Ying H Qiao C Yang X Lin X. A case report of 2 sirolimus-related deaths among infants with kaposiform hemangioendotheliomas. Pediatrics. (2018) 141(Suppl. 5):S425–9. 10.1542/peds.2016-2919 29610165

[34] Bush R Johns F Acharya R Upadhyay K. Mild COVID-19 in a pediatric renal transplant recipient. Am J Transplant. (2020) 20:2942–5. 10.1111/ajt.16003 32406181 PMC7272978

[35] Omarjee L Janin A Perrot F Laviolle B Meilhac O Mahe G. Targeting T-cell senescence and cytokine storm with rapamycin to prevent severe progression in COVID-19. Clin Immunol. (2020) 216:108464. 10.1016/j.clim.2020.108464 32405269 PMC7217787

[36] Meziyerh S Zwart TC van Etten RW Janson JA van Gelder T Alwayn IPJ. Severe COVID-19 in a renal transplant recipient: a focus on pharmacokinetics. Am J Transplant. (2020) 20:1896–901. 10.1111/ajt.15943 32337790 PMC7267503

